# A change in social participation affects cognitive function in middle-aged and older Chinese adults: analysis of a Chinese longitudinal study on aging (2011–2018)

**DOI:** 10.3389/fpubh.2024.1295433

**Published:** 2024-02-02

**Authors:** Xuyang Li, Wenyan Xu

**Affiliations:** ^1^School of Public Health, Wuhan University, Wuhan, China; ^2^Jiangxi Provincial People's Hospital, The First Affiliated Hospital of Nanchang Medical College, Nanchang, China

**Keywords:** cognitive function, social participation, longitudinal study, aging, China

## Abstract

**Background:**

One of the biggest challenges facing older adults is cognitive decline and social participation has always been considered a protective factor. However, it is not clear whether social participation predicts cognitive function in this population, rather than depressive symptoms, self-reported health, and activities of daily life, with sufficient capacity to detect unique effects.

**Methods:**

This study included adults aged 45 and above in China (*N* = 5,258) who participated in a large national older adult health survey and provided data from 2011, 2013, 2015, and 2018. The unique associations between the predictors of social participation and cognitive function over time and context were evaluated in the Latent Growth Model (LGM).

**Results:**

Among the 5,258 participants in our study, an overall cognitive decline was observed. Social participation predicts two dimensions of cognitive function, with a degree of impact comparable to depressive symptoms, self-reported health, and activities of daily life. Among them, social participation exhibits a noteworthy prognostic impact on episodic memory during the same period. The regression coefficient is approximately 0.1 (*p* < 0.05) after controlling other mixed variables (depressive symptoms, self-reported health, and activities of daily life). In contrast, social participation is also a significant predictor of mental intactness in the same period, with a regression coefficient of 0.06 (*p* < 0.05), even if all mixed variables are controlled.

**Conclusion:**

Over time, the correlation strength of social participation is comparable to other recognized cognitive function prediction indicators, indicating that promoting social participation among middle-aged and older Chinese adults is a meaningful way to improve cognitive function degradation, which has important policy and practical significance.

## 1 Introduction

In 2020, the population of China aged 65 and above exceeded 190.6 million, accounting for 13.5% of the total population (1). According to official forecast data, the olde adult population aged 65 and above in China will reach approximately 330 million by 2050 (1, 2). With the deepening of population aging, the scale of cognitive dysfunction in older adults is also increasing, with a prevalence rate of 15.5% (3). Cognitive function refers to the advanced function of the cerebral cortex in processing and understanding received information, determined by various neural, psychological, and emotional factors, and is crucial for memory storage, emotional control, and decision-making (4, 5). In the process of aging, once cognitive impairment becomes severe, it can develop into neurodegenerative diseases such as Alzheimer’s disease, seriously reducing the quality of life of older adults. Furthermore, the demand for treatment and rehabilitation will also increase significantly, and the intervention of family and social resources is urgently needed (6).

Social participation refers to the provision of accompanying activities for individuals to interact with others in society (7, 8). It is an important contributor to the health and well-being outcomes of older adults and one of the three pillars of the World Health Organization’s (WHO) policy to promote active aging societies. Due to its popularity, low cost, and expected benefits for mental health, it has attracted much attention (9). Previous studies have shown that the social participation rate of older adults in China ranges from 45.90 to 53.32% (10–14). There are two main theories about the relationship between social participation and cognitive function: the use and disuse theory suggests that a decrease in social participation leads to cognitive decline of the brain, while a stimulated mind can improve cognitive function (15). Therefore, cognitive decline can be prevented in old age through participation in a variety of mental and physical activities. Especially when cognitive abilities begin to decline, external stimulation is more important for older adults. Social identity theory holds that social participation can enable individuals to acquire the identity of a being member of a certain group (16, 17). This identity influences the individual’s values, emotions, and health-related behaviors and provides the individual with social resources and support that can help improve the individual’s cognitive function.

Some empirical studies provide support for the above theory. The findings of James et al. suggest that social activities are related to cognition throughout life, especially among older adults (18). For example, traditional Chinese social participation activities such as Tai Chi and Qing Kung can help maintain cognitive function among older adults in China (19–21). Moreover, a population-based longitudinal study conducted by Glei showed that participating in volunteer activities and socializing with friends can help maintain the cognitive function of older adults (22). In addition, there have been a few observational studies and studies based on pathophysiological mechanisms, indicating a possible reverse association between social participation and cognitive function. For example, a pathologically controlled study suggests that individuals in the preclinical stages of long-term dementia might be more likely to avoid participating in cognitive activities than their healthy peers (23). An Israeli study of adults also found a similar bidirectional association result (24). However, the current academic mainstream studies still regard the decrease in social activity participation as the cause rather than the result of cognitive decline (25), especially given the low proportion and single form of social participation of older adults in China, the inadequate construction of the community for older adults, and the increasing proportion of older adults living alone year by year (26, 27). Therefore, it is necessary to identify the impact of social participation over time on the cognitive function of middle-aged and older Chinese adults.

In previous studies, many factors have been associated with cognitive decline in older adults (28). Although most existing studies focus on the genetic and biological determinants, these findings create more space for the role of social factors in the decline of cognitive function in older adults. Existing research has reached a consensus on alleviating cognitive decline, indicating that physical health, depressive symptoms, and activities of daily life (ADL) are important cognitive resources in the lives of older adults (29–31). Social participation, depressive symptoms, self-rated health, and ADL may not be the only predictors of cognitive function. On the contrary, they have a common path. For example, a decline in health may lead to an increase in depressive symptoms, which in turn affects cognitive function (14). Social participation can also play a role alongside other predictors. For example, people with poor health may be unable to participate in social activities, and people with more severe depressive symptoms may reduce going out to socialize. However, participating in social activities also provides older adults with a unique opportunity to engage in cognitive tasks beyond the influence of health, depressive symptoms, and ADL to maintain cognitive function (32, 33).

The social structure background in China is different from that in developed countries. In family life, older adults may need to play both the roles of children and grandparents, which means that most of them need to take on the responsibility of taking care of their older adults parents and young grandchildren at the same time. Their focus has shifted from work and family to personal life and family. In terms of time allocation, older adults may face a choice between personal life and family care and may even sacrifice a more autonomous lifestyle due to the responsibility of family care, so the opportunities for Chinese older adults to participate in social activities are very limited (34). Therefore, our paper has two main purposes. Firstly, we use the sample data of middle-aged and older Chinese adults to assess the strength of the correlation between social participation and cognitive function over time. Secondly, we attempt to determine whether social participation is independently correlated with cognitive function. This study hypothesizes that after controlling for three recognized predictors of cognitive decline, i.e., depressive symptoms, self-reported health, and ADL, social participation would uniquely predict cognitive function. Finally, we explore whether the correlation between social participation and cognitive function differs from other predictors.

## 2 Methods

### 2.1 Sample

This sample was derived from the China Health and Retirement Longitudinal Study (CHARLS), which surveyed people aged 45 and above in 150 county-level units and 450 communities in 28 provinces of China in 2011, 2013, 2015, and 2018. After excluding all missing indicators included in the baseline periods of 2011, 2013, 2015, and 2018, 5,258 participants aged 45 and above were included in this study. The longitudinal data analysis of the cognitive function of middle-aged and older Chinese adults in relation to their social participation was conducted using four waves of data in 2011 (T1), 2013 (T2), 2015 (T3), and 2018 (T4).

### 2.2 Measurements

#### 2.2.1 Dependent variable – cognitive function

The calculation of cognitive function can be divided into episodic memory and mental intactness (35, 36). The calculation of episodic memory scores is based on the word recall test, which is divided into immediate recall and delayed recall. Immediate recall refers to the examiner randomly reading out 10 words and then asking participants to immediately recall as many words as possible, scoring the correct number of words recalled (21). Delayed recall refers to 10 min later when participants are asked to recall the same word list (36). The score of episodic memory refers to the average number of words in both immediate and delayed recall, ranging from 0 to 10 (37). Mental intactness aims to capture the mental intactness or state of individuals. In CHARLS, mental intactness problems include 7 consecutive subtractions from 100 (up to 5 times), date (month, day, year), day of the week, season, and cross pentagon replication tests. The answers to these questions are summarized into a mental intactness score of 0 ~ 11 (36).

#### 2.2.2 Independent variables – social participation

In CHARLS, we evaluated 10 social activities of older adults over the past month, including communicating with friends, stock investment, and using the Internet. The frequency of each activity was categorized as never, irregular, almost weekly, or almost daily, with a score of 0 ~ 3. These were used to calculate a comprehensive social participation score ( $\mathrm{Social}\;\mathrm{participatio}n_{ij}=\sum \mathrm{Social}\;\mathrm{activiti}e_{ij}*\mathrm{frequenc}y_{ij}$ ). Overall, the total score of social participation ranged from 0 to 24. The higher the score, the higher the level of social participation (36, 38, 39).

#### 2.2.3 Covariates

##### 2.2.3.1 Depressive symptoms

The Center for Epidemiological Studies Depression Scale (CES-D) was adopted, which was composed of 10 items (40). The answer options were never, sometimes or rarely, often, and always, with a score of 0 ~ 3. The total score of depressive symptoms ranged from 0 to 30. The higher the score, the more severe the depressive symptoms.

##### 2.2.3.2 Self-reported health

The respondents were asked “How would you rate your health?,” and the response options ranged from “very good” to “poor.” While the response options for this question in 2018 were slightly different from the data of the previous three phases, it was treated as a continuous variable, on a scale of 1 to 5. The higher the score, the worse the self-reported health.

##### 2.2.3.3 Activities of daily life

Respondents were asked if they had difficulties in six lifestyle habits (dressing, bathing, eating, getting up, going to the bathroom, and doing things themselves) or five instrumental activities (doing housework, cooking, shopping, managing finances, and taking medicine). The answer scale included four options: (1) No, I have no difficulty; (2) I have difficulties but I can still do it; (3) Yes, I have difficulties and need help; (4) I cannot do it (41). The subjects of this study were middle-aged and older adults, and most of them could independently complete the above behaviors. Therefore, this variable was considered a binary variable. If they reported having difficulty completing any item, it was defined as limited activities of daily life (ADL). Each ADL or instrumental activity of daily living (IADL) item had a score of 0 or 1, with a total score of up to 11 points. The higher the total score of ADL, the poorer the functional health (31, 42, 43).

##### 2.2.3.4 Other covariates

Other covariates include demographic variables in the baseline period, including age, gender, and education level.

### 2.3 Statistical analysis

In this study, we first conducted descriptive statistics of each variable and calculated the correlation coefficient between them, examining the stability of interrelationships of other variables such as cognitive function and social participation in middle-aged and older Chinese adults.

**Figure 1:**
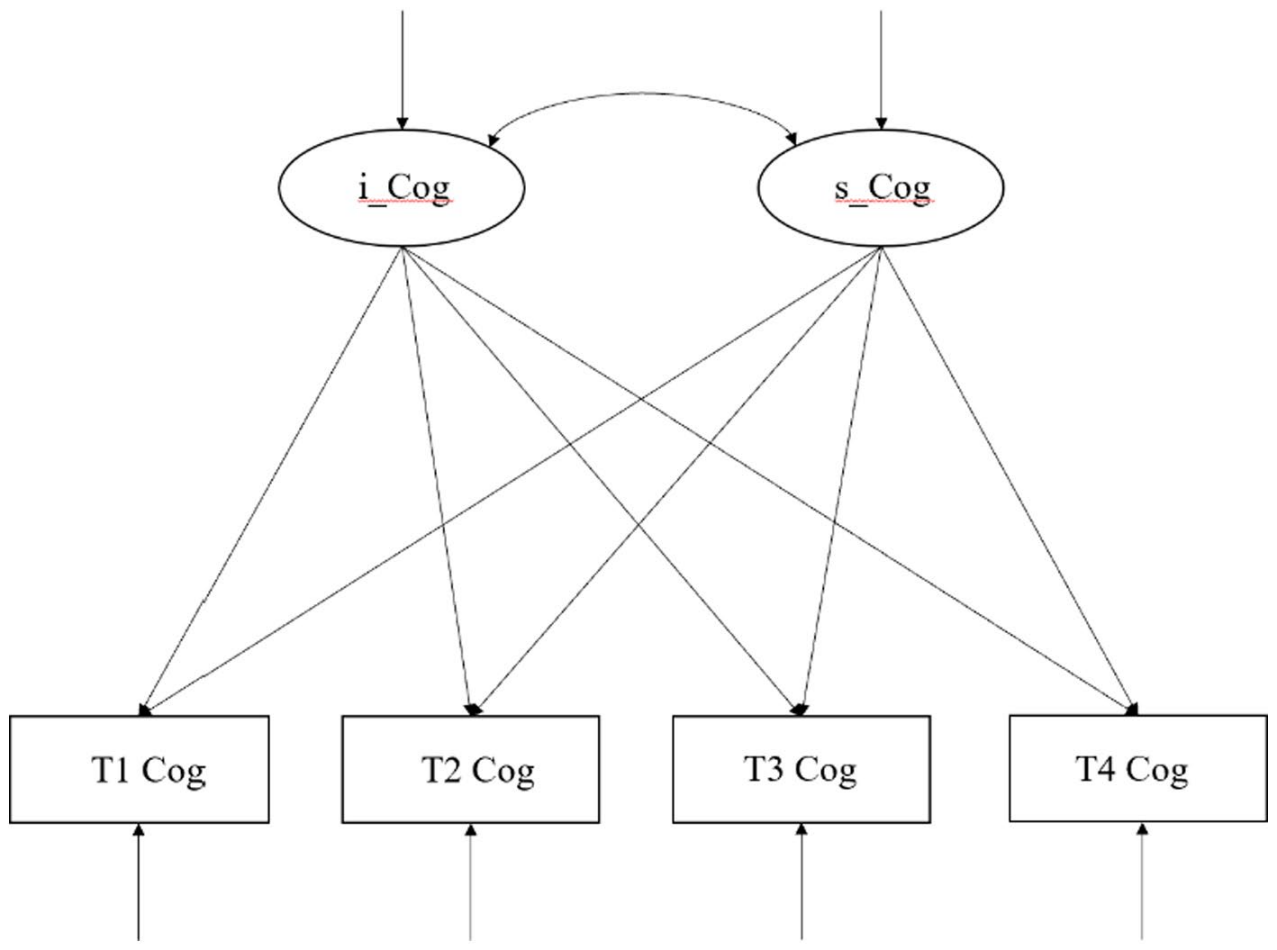
A linear unconditional latent growth model of cognitive function of middle-aged and older adults in China.

**Figure 2:**
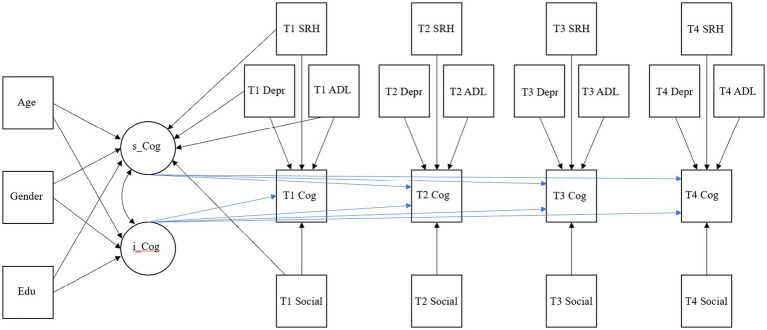
A conceptual model for predicting cognitive function in middle-aged and older adults through social participation. The model was suitable for episodic memory and mental intactness, including time-varying and invariant covariates. It regressed the explicit variables and latent parameters of cognitive function, respectively, to provide a comparative analysis of their impact on cognitive function. For T1, T2, T3, and T4, the parameter estimates for calculating the slope of cognitive function were constrained to 1, 2, 3, and 4, respectively. The residuals of endogenous variables are not specified but were included in the final model. All paths represent standardized model values. Cog, cognitive function; dep, depressive symptoms; Social, social participation; SRH, self-reported health; ADL, activities of daily life; Edu, education; s_ and i_, the slope and intercept of the described structure.

Then, we constructed an unconditional LGM to examine the trajectory of cognitive function changes among middle-aged and older Chinese adults and whether there were significant individual differences in the initial level and development rate of cognitive function. On this basis, we ultimately constructed a conditional LGM and added the age, gender, and education level in the baseline period, which are three constant factors that do not change with time, to investigate whether there were demographic differences in cognitive function. The four time-varying factors of social participation, depressive symptoms, self-reported health, and ADL were added to investigate whether the cognitive function was affected over time by social participation, depressive symptoms, self-reported health, and ADL. Statistical analysis was conducted using Mplus 8.0 software, with a significance level of 0.05. The conceptual analysis model is shown in Figures 1, 2.

## 3 Results

### 3.1 Study characteristics

The average age of the participants in T1 was 57.078 years (SD = 8.019). Most of them were men, accounting for 56.999%. The education level of the cohort was mainly junior high school or below, accounting for 85.299%. The mean social participation scores at T1, T2, and T3 increased from 1.620 to 2.118, with a slight decline in T4. From T1 to T4, the mental intactness of cognitive function showed a declining trend, with an average of 7.857 to 6.739. In addition, the ADL showed an increasing trend, from 0.502 to 0.816 (see Table 1). The Pearson’s test obtained the correlation matrix for all study variables (see Table 2). There was a significant positive correlation between social participation and cognitive function among middle-aged and older adults at different times. Depressive symptoms, self-rated health, and ADL showed a negative correlation.

**Table 1:** Descriptive statistics of respondents on key variables.

*N* = 5,258	T1	T2	T3	T4
Episodic memory	3.718 ± 1.641	3.782 ± 1.702	3.523 ± 1.730	4.215 ± 2.284
Mental intactness	7.857 ± 2.818	7.655 ± 2.938	7.512 ± 2.942	6.739 ± 2.959
Social participation	1.620 ± 2.191	2.089 ± 2.548	2.118 ± 2.727	1.800 ± 2.521
Depressive symptoms	7.931 ± 6.148	7.500 ± 5.611	7.618 ± 6.221	8.358 ± 6.383
Self-reported health	3.826 ± 0.898	3.797 ± 0.884	3.823 ± 0.920	2.996 ± 0.992
ADL	0.502 ± 1.314	0.508 ± 1.338	0.665 ± 1.536	0.816 ± 1.804
Age	57.078 ± 8.019			
Gender	56.999% men			
Education	85.299% Middle school and below			

Data are means ± standard deviations.

**Table 2:** Correlation matrix for variables.

Sample																											
	1	2	3	4	5	6	7	8	9	10	11	12	13	14	15	16	17	18	19	20	21	22	23	24	25	26	27
EM T1 (1)	1																										
EM T2 (2)	0.400**	1																									
EM T3 (3)	0.437**	0.483**	1																								
EM T4 (4)	0.406**	0.472**	0.514**	1																							
MI T1 (5)	0.344**	0.319**	0.324**	0.415**	1																						
MI T2 (6)	0.311**	0.379**	0.368**	0.446**	0.562**	1																					
MI T3 (7)	0.314**	0.360**	0.398**	0.467**	0.579**	0.598**	1																				
MI T4 (8)	0.315**	0.354**	0.378**	0.522**	0.560**	0.586**	0.613**	1																			
Soc T1 (9)	0.179**	0.160**	0.164**	0.159**	0.157**	0.149**	0.149**	0.159**	1																		
Soc T2 (10)	0.175**	0.210**	0.189**	0.196**	0.184**	0.202**	0.183**	0.181**	0.340**	1																	
Soc T3 (11)	0.159**	0.178**	0.190**	0.191**	0.157**	0.164**	0.173**	0.165**	0.329**	0.385**	1																
Soc T4 (12)	0.173**	0.197**	0.202**	0.234**	0.157**	0.165**	0.160**	0.187**	0.260**	0.324**	0.377**	1															
Dep T1 (13)	−0.218**	−0.176**	−0.167**	−0.178**	−0.258**	−0.231**	−0.219**	−0.199**	−0.111**	−0.119**	−0.105**	−0.091**	1														
Dep T2 (14)	−0.153**	−0.172**	−0.145**	−0.154**	−0.202**	−0.225**	−0.184**	−0.175**	−0.085**	−0.105**	−0.097**	−0.090**	0.510**	1													
Dep T3 (15)	−0.154**	−0.149**	−0.183**	−0.168**	−0.230**	−0.216**	−0.229**	−0.196**	−0.071**	−0.101**	−0.110**	−0.093**	0.481**	0.538**	1												
Dep T4 (16)	−0.129**	−0.138**	−0.140**	−0.188**	−0.199**	−0.205**	−0.202**	−0.224**	−0.087**	−0.107**	−0.102**	−0.092**	0.424**	0.499**	0.518**	1											
SRH T1 (17)	−0.133**	−0.119**	−0.110**	−0.103**	−0.146**	−0.123**	−0.118**	−0.118**	−0.081**	−0.078**	−0.091**	−0.102**	0.364**	0.293**	0.252**	0.249**	1										
SRH T2 (18)	−0.103**	−0.111**	−0.108**	−0.093**	−0.121**	−0.115**	−0.113**	−0.095**	−0.043**	−0.091**	−0.095**	−0.111**	0.293**	0.370**	0.303**	0.294**	0.421**	1									
SRH T3 (19)	−0.099**	−0.108**	−0.115**	−0.095**	−0.114**	−0.090**	−0.112**	−0.093**	−0.060**	−0.066**	−0.102**	−0.104**	0.277**	0.322**	0.365**	0.301**	0.383**	0.459**	1								
SRH T4 (20)	−0.093**	−0.100**	−0.113**	−0.119**	−0.118**	−0.104**	−0.122**	−0.116**	−0.068**	−0.092**	−0.101**	−0.124**	0.271**	0.299**	0.310**	0.376**	0.367**	0.432**	0.473**	1							
ADL T1 (21)	−0.142**	−0.131**	−0.141**	−0.150**	−0.206**	−0.180**	−0.156**	−0.149**	−0.091**	−0.104**	−0.097**	−0.087**	0.330**	0.222**	0.253**	0.215**	0.243**	0.187**	0.173**	0.202**	1						
ADL T2 (22)	−0.119**	−0.133**	−0.134**	−0.148**	−0.155**	−0.177**	−0.148**	−0.158**	−0.069**	−0.073**	−0.091**	−0.085**	0.262**	0.340**	0.293**	0.262**	0.210**	0.247**	0.212**	0.247**	0.420**	1					
ADL T3 (23)	−0.124**	−0.148**	−0.149**	−0.171**	−0.185**	−0.185**	−0.209**	−0.190**	−0.079**	−0.102**	−0.106**	−0.104**	0.278**	0.294**	0.386**	0.285**	0.216**	0.243**	0.284**	0.297**	0.429**	0.507**	1				
ADL T4 (24)	−0.136**	−0.145**	−0.147**	−0.192**	−0.160**	−0.181**	−0.184**	−0.224**	−0.102**	−0.104**	−0.114**	−0.115**	0.262**	0.262**	0.303**	0.345**	0.214**	0.208**	0.228**	0.355**	0.403**	0.454**	0.572**	1			
Age (25)	−0.195**	−0.234**	−0.281**	−0.330**	−0.106**	−0.113**	−0.132**	−0.150**	−0.098**	−0.122**	−0.142**	−0.150**	0.075**	0.049**	0.046**	0.007	0.060**	0.037**	0.057**	0.100**	0.153**	0.135**	0.168**	0.194**	1		
Gender (26)	−0.028*	−0.048**	−0.001	−0.048**	−0.265**	−0.245**	−0.243**	−0.214**	−0.071**	−0.057**	−0.066**	−0.025	0.166**	0.178**	0.184**	0.189**	0.101**	0.081**	0.084**	0.061**	0.074**	0.066**	0.093**	0.087**	−0.082**	1	
Edu (27)	0.218**	0.246**	0.248**	0.269**	0.248**	0.263**	0.260**	0.254**	0.196**	0.229**	0.188**	0.223**	−0.153**	−0.114**	−0.125**	−0.125**	−0.112**	−0.086**	−0.081**	−0.091**	−0.083**	−0.072**	−0.087**	−0.088**	−0.104**	−0.115**	1

EM, episodic memory; MI, mental intactness; Dep, depressive symptoms; Soc, social participation; SRH, self-reported health; ADL, activities of daily life; Edu, education.

**p < 0.01; *p < 0.05.

### 3.2 Unconditional LGM

To test whether the developmental trajectory of cognitive function in middle-aged and older adults is linear or nonlinear, we built linear and nonlinear unconditional LGM, respectively. From the fitting index, it can be seen that the linear unconditional LGM has a better fitting effect on the data than the nonlinear unconditional LGM, indicating that the development of cognitive function (episodic memory and mental intactness) in middle-aged and older adults showed a linear trend from T1 to T4.

Therefore, first, we built the unconditional linear LGM of episodic memory. According to the common evaluation standard of fitting index, the model fitted well. The model fitting indexes were: *χ*^2^ = 543.373, df = 5, *p* < 0.001, TLI = 0.894, CFI =0.873, SRMR = 0.052. Next, we built the unconditional linear LGM of mental intactness. The model fitting result of mental intactness was good, *χ*^2^ = 172.590, df = 5, p < 0.001, TLI = 0.976, CFI =0.980, SRMR = 0.024. After establishing the basic LGM, we introduced relevant time-varying variables (social participation, depressive symptom, self-reported health, and ADL) that predicted the cognitive variables in T1, T2, T3, and T4, and non-time-varying covariates (baseline age, gender, and education level) to predict the slope and intercept of episodic memory and mental intactness in the two models. Finally, we included direct correlations between social participation, depressive symptoms, self-reported health, and ADL at T1 to predict the slope of cognitive measurement.

### 3.3 LGM included covariates

#### 3.3.1 Episodic memory

The model results of the final episodic memory fitted well, *χ*^2^ (35, *N* = 5,258) =226.847, *p* < 0.001, CFI = 0.975, RMSEA = 0.024. Firstly, age, as a covariate that does not change with time, had a significant predictive effect on the intercept and slope of episodic memory [*B* = -0.250, (−0.293, −0.206), *B* = -0.519, (−0.651, −0.386)]. Education level also predicted the intercept [*B* = 0.288, (0.244, 0.332)] and slope [*B* = 0.213, (0.121, 0.305)] of episodic memory. Gender had no predictive effect on the intercept and slope of episodic memory, and the *p*-values were not significant and not statistically significant.

Next, we investigated the strength of the time-varying covariates that predict episodic memory over time: social participation, depressive symptoms, self-reported health, and ADL. Both social participation and depressive symptoms significantly predicted episodic memory at all time points, T1, T2, T3, and T4. However, self-reported health and ADL could not significantly predict episodic memory at T1 and T3, respectively, with *p* > 0.05, which was not statistically significant. In addition, social participation, depressive symptoms, and ADL independently predicted the slope of episodic memory. For example, lower social participation and ADL and more severe depressive symptoms uniquely predicted the sharp decline of episodic memory function (44). See Table 3 below for the complete standardized model results. The comparison of the standardized strength of social participation and depressive symptoms’ association with episodic memory revealed that competing predictors were relatively comparable, in terms of context and predicting the slope of episodic memory (44).

**Table 3:** Parameter estimates for LGM parameters, model fit, and regression coefficients predicting episodic memory.

Parameter	Mean	Variance	
Intercept	6.025***	0.840***	
Slope	3.861***	0.439**	
Fit statistics			
H1 log-likelihood	−38938.300		
No. of parameters	35		
*χ* ^2^	226.847		
RMSEA	0.024		
CFI	0.975		
Intercept of episodic memory	*B*	95%CI	β
Age	−0.250***	[−0.293, −0.206]	−0.027***
Gender	0.019	[−0.024, 0.062]	0.034
Education	0.288***	[0.244, 0.332]	0.709***
Slope of episodic memory			
Age	−0.519***	[−0.651, −0.386]	−0.015***
Gender	−0.003	[−0.084, 0.078]	−0.001
Education	0.213***	[0.121, 0.305]	0.135***
Social participation at T1	0.228***	[0.139, 0.317]	0.023***
Depressive symptoms at T1	−0.203***	[−0.297, −0.108]	−0.007***
Self-reported health at T1	0.018	[−0.062, 0.098]	0.005
ADL at T1	−0.114**	[−0.199, −0.030]	−0.020**
Episodic memory at T1			
Social participation at T1	0.112***	[0.087, 0.137]	0.083***
Depressive symptoms at T1	−0.144***	[−0.172, −0.116]	−0.038***
Self-reported health at T1	−0.015	[−0.038, 0.009]	−0.027
ADL at T1	−0.044**	[−0.071, −0.017]	−0.054**
Episodic memory at T2			
Social participation at T2	0.081***	[0.059, 0.104]	0.054***
Depressive symptoms at T2	−0.086***	[−0.111, −0.060]	−0.026***
Self-reported health at T2	−0.024**	[−0.041, −0.008]	−0.046**
ADL at T2	−0.025*	[−0.049, −0.001]	−0.031*
Episodic memory at T3			
Social participation at T3	0.028*	[0.007, 0.049]	0.018*
Depressive symptoms at T3	−0.075***	[−0.099, −0.050]	−0.021***
Self-reported health at T3	−0.061***	[−0.077, −0.045]	−0.116***
ADL at T3	0.007	[−0.017, 0.031]	0.008
Episodic memory at T4			
Social participation at T4	0.087***	[0.065, 0.109]	0.078***
Depressive symptoms at T4	−0.090***	[−0.115, −0.065]	−0.032***
Self-reported health at T4	0.035**	[0.012, 0.058]	0.079**
ADL at T4	−0.050***	[−0.075, −0.025]	−0.062***

#### 3.3.2 Mental intactness

The final model results of mental intactness fitted well, *χ*^2^ (35, *N* = 5,258) =243.244, *p* < 0.001, CFI = 0.982, RMSEA = 0.026. First, age, a covariate that does not change over time, had a significant predictive effect on the intercept and slope of mental intactness, *B* = -0.107, [−0.140, 0.075], *B* = -0.155, [−0.267, −0.042]. Gender also predicted intercept [*B* = -0.315, (−0.346, −0.283)] and slope [*B* = 0.154, (0.042, 0.266)] of mental intactness. In contrast, the intercept of education level in predicting mental intactness [*B* = 0.262, (0.230, 0.295)] cannot predict the slope of mental intactness, and the value of p is not significant, which is not statistically significant.

Next, we examine the strength of time-varying covariates; social participation, depressive symptoms, self-reported health, and ADL predicted mental intactness over time. Social participation, depressive symptoms, and ADL significantly predicted mental intactness at four time points. In addition, social participation and depressive symptoms can independently predict the slope of mental intactness. For example, lower social participation and higher depressive symptoms can independently predict the sharp decline of mental intactness. See Table 4 for the complete standardized model results. Comparisons of the standardized strength of social participation, depressive symptoms, and ADL’s association with mental intactness reveal that competing predictors were relatively comparable in terms of context and predicting the slope of mental intactness.

**Table 4:** Parameter estimates for LGM parameters, model fit, and regression coefficients predicting mental intactness.

Parameter	Mean	Variance	
Intercept	5.918***	0.801***	
Slope	−1.300*	0.757***	
Fit statistics			
H1 log-likelihood	−47148.172		
No. of parameters	35		
*χ* ^2^	243.244		
RMSEA	0.026		
CFI	0.982		
Intercept of mental intactness	*B*	95%CI	β
Age	−0.107***	[−0.140, 0.075]	−0.027***
Gender	−0.315***	[−0.346, −0.283]	−1.260***
Education	0.262***	[0.230, 0.295]	1.469***
Slope of mental intactness			
Age	−0.155**	[−0.267, −0.042]	−0.005**
Gender	0.154**	[0.042, 0.266]	0.075**
Education	0.034	[−0.066, 0.135]	0.023
Social participation at T1	0.252***	[0.119, 0.385]	0.028***
Depressive symptoms at T1	−0.272***	[−0.419, −0.124]	−0.011***
Self-reported health at T1	−0.051	[−0.157, 0.056]	−0.014
ADL at T1	−0.067	[−0.177, 0.044]	−0.012
Mental intactness at T1			
Social participation at T1	0.060***	[0.037, 0.083]	0.077***
Depressive symptoms at T1	−0.116***	[−0.142, −0.090]	−0.053***
Self-reported health at T1	−0.031**	[−0.053, −0.010]	−0.099**
ADL at T1	−0.086***	[−0.110, −0.061]	−0.184***
Mental intactness at T2			
Social participation at T2	0.064***	[0.044, 0.084]	0.073***
Depressive symptoms at T2	−0.095***	[−0.118, −0.072]	−0.049***
Self-reported health at T2	−0.009	[−0.024, 0.006]	−0.031
ADL at T2	−0.066***	[−0.087, −0.044]	−0.142***
Mental intactness at T3			
Social participation at T3	0.048***	[0.028, 0.067]	0.051***
Depressive symptoms at T3	−0.064***	[−0.087, −0.041]	−0.030***
Self-reported health at T3	0.018*	[0.004, 0.031]	0.056*
ADL at T3	−0.073***	[−0.095, −0.051]	−0.137***
Mental intactness at T4			
Social participation at T4	0.037***	[0.017, 0.058]	0.044***
Depressive symptoms at T4	−0.057***	[−0.080, −0.034]	−0.027***
Self-reported health at T4	0.008	[−0.013, 0.030]	0.025
ADL at T4	−0.081***	[−0.104, −0.059]	−0.134***

## 4 Discussion

This study analyzed the relationship between social participation and cognitive function using four-wave longitudinal data of 5,258 middle-aged and older Chinese adults (45). After controlling for depressive symptoms, self-reported health, and ADL, we examined the different intensities of frequency and duration of social participation and cognitive function of this population (episodic memory and mental intactness). The results showed that the higher the level of social participation, the higher the score of cognitive function. Consistent with previous studies, social participation can reduce the risk of cognitive decline (18, 46, 47). In addition, this study also found that social participation during the baseline period can predict changes in cognitive function over time (48). At the same level, factors such as social participation, depressive symptoms, self-reported health, and ADL also affect the cognitive function of middle-aged and older Chinese adults.

Consistent with previous research results, social participation and cognitive function (episodic memory and mental intactness) showed a significant positive correlation over time, which also confirms the first hypothesis of this study. According to the theory of “cognitive reserve,” the participation of older adults in intellectual, social, and physical activities can optimize the memory-related brain regions or brain networks, resulting in less impact on memory function in middle-aged and older Chinese adults, thus preventing the occurrence of dementia (49). The stress theory suggests that the body needs attention to break down stress, which can compete for cognitive resources (50). In addition, stress may be related to the dysregulation of cortisol and inflammatory cytokines, which can lead to a series of negative health outcomes, such as cognitive decline, when stress levels are elevated (51–53). Furthermore, social participation has a protective effect on cognitive function by regulating stress to eliminate the negative effects of stressful events (54). The vascular theory suggests that active social participation reduces the risk of vascular diseases and their risk factors (55), and vascular diseases or vascular risk factors are closely related to the progression of cognitive disorders such as Alzheimer’s disease; therefore, social participation can affect the occurrence of cognitive decline by affecting vascular diseases (56).

Inconsistent with previous studies on the single impact of social participation on cognitive function, this study suggests that low social participation, high depressive symptoms, and ADL at T1 can uniquely predict cognitive decline in middle-aged and older Chinese adults, further supporting the cognitive enrichment hypothesis (44). First of all, the results of this study found that those who rated themselves as healthier had better cognitive function. Consistent with previous research results, individual health not only affects cognitive function over time, but also, the higher the degree of self-perceived health, the better their cognitive abilities (57, 58). These findings support the Strength and Vulnerability Integration (SAVI) model, which suggests that, due to the increased physical vulnerability of middle-aged and older adults, they may be more vulnerable to stress and suffer more severe cognitive and emotional consequences than younger adults (59). Compared with middle-aged and older adults with good health, people with poor health are more likely to suffer from stress, resulting in impaired cognitive function (60). In addition, other studies have found that cognitive performance and health may have a common cause; if the effects of this common cause become increasingly pronounced with age, it could explain the current findings. For example, the aforementioned vascular theory suggests that vascular risk factors (such as hypertension, coronary heart disease, and dyslipidemia) become more common with age, and they are related to cognitive impairment disorders such as Alzheimer’s disease (61, 62).

Second, numerous studies have emphasized the possibility of multiple mechanisms that explain the decline in cognitive ability caused by depressive symptoms (63, 64). The results of this study enrich existing research, showing that depressive symptoms in middle-aged and older Chinese adults predict cognitive decline (episodic memory and mental intactness) over time (63, 65). The occurrence factors of depressive symptoms in middle-aged and older Chinese adults are complex and are influenced by heredity, physical diseases, and environment. Persistent stress events are one of the important factors leading to depression, and they also have the potential to damage cognitive function in older adults. The impairment of episodic memory and mental intactness is the most common manifestation, but the molecular mechanism involved is still unclear. Based on previous literature, one possible mechanism is that depressive symptoms may be a psychological response to a decline in self-awareness, a decline in interest in anything, and a reluctance to communicate with the outside world. People with depressive symptoms also have greater impairments in attention and language function, interfering with daily activities and resulting in an inability to exert greater effort when carrying out tasks (66). In terms of physiological mechanism, hippocampal volume has been linked to language and spatial memory function (66). Depressive symptoms may lead to dysregulation of the hypothalamic pituitary adrenal axis, leading to prolonged hypercortisolemia, hippocampal atrophy, and impaired language and spatial memory functions (66, 67). In addition, previous studies on the genetic susceptibility of depressive symptoms have shown that patients with depressive symptoms cannot bear the excessive and severe living demands, leading to chronic stress and potentially interfering with cognitive functions such as attention, memory, and information processing (68, 69).

Third, the measurement of cognitive function in older adults through episodic memory mainly focuses on memory ability, while mental intactness involves other mental abilities such as reading and computing. Previous studies have shown that the daily activities of middle-aged and older Chinese adults are associated with a slowdown in brain atrophy, which may support the decline of the executive control processes and memory function of older adults (70–72). Juan Wang et al. also found that daily activities are associated with cognitive function in older adults, which is also consistent with our research (31). The reason may be that limited daily behavior ability can cause older adults to be unable to take care of themselves completely, resulting in a reduced range of activities, decreased interaction with the outside world, and less information obtained. Due to the lack of sufficient information stimulation, the brain cannot always be active, which leads to the deterioration of brain function and increases the risk of cognitive function damage (73).

Therefore, when formulating social health interventions targeting middle-aged and older Chinese adults, we should pay attention to their physical and emotional aspects. Firstly, families, communities, governments, and other social organizations should consider how to take targeted measures to reduce the negative emotions of this population, enhance their openness and enthusiasm toward life, and prevent “dementia” caused by “depression.” Secondly, according to the enrichment hypothesis, communities, governments, and other social organizations should formulate corresponding activity plans to make social activities attractive, stimulating, and enjoyable, creating more fun for retired older adults (74). Middle-aged and older Chinese adults should be encouraged to actively participate in social interactions and increase their channels of social participation. The establishment of community mutual aid organizations, the promotion of the “Time Bank” project, and the protection of the cognitive function of older adults should also be encouraged..

This study also has some limitations. First, due to the bias of the self-report method, the prevalence of sensitive issues such as cognitive function and depressive symptoms in China may be underestimated, and there is a certain recall bias. Our research results show that the self-report method may also be considered an advantage and may be a necessary preliminary method to lay the foundation for subsequent perceptual intervention trials to examine these mechanisms through objective mechanisms. Second, this study found that higher social participation was associated with better cognitive function. However, reverse causation is also possible. Similar to previous studies, there is still insufficient evidence for reverse causal relationships between social participation and cognitive function in this study (18, 36, 74). Third, the current study is limited to general indicators of social activities. The existing data do not include specific physiological and psychological information about social activities. In the future, we can use data such as the specific psychological benefits derived from participating in social activities to elaborate more on the potential mechanisms.

## 5 Conclusion

This study provides evidence for the mechanisms of the influence of social participation on cognitive function among middle-aged and older Chinese adults. Over time, social participation predicts episodic memory and mental intactness in middle-aged and older Chinese adults, independent of the effects of depressive symptoms, self-reported health, and ADL, and to the same extent as these competing predictors and cognitive ability. Therefore, interventions that target cognition by participating in social activities are promising and may be as effective or more effective than treatments that target other predictors of cognitive decline, such as depressive symptoms, self-reported health, and ADL.

## Data availability statement

The datasets presented in this study can be found in online repositories. The names of the repository/repositories and accession number (s) can be found below: the original databases of this study are available from the online site: https://charls.charlsdata.com/index/zh-cn.html.

## Ethics statement

The studies involving humans were approved by the Approval for the original CHARLS was obtained from the Biomedical Ethics Review Committee of Peking University (IRB00001052-11015), and all participants signed informed consent at the time of participation. The studies were conducted in accordance with the local legislation and institutional requirements. The participants provided their written informed consent to participate in this study.

## Author contributions

XL: Conceptualization, Data curation, Methodology, Software, Writing – original draft, Writing – review & editing. WX: Writing – review & editing.
